# 914. Incidence of Herpes Zoster and Postherpetic Neuralgia and Herpes Zoster Vaccination in a United States Administrative Claims Database

**DOI:** 10.1093/ofid/ofad500.959

**Published:** 2023-11-27

**Authors:** Purva Jain, Alan Embry, Brent Arakaki, Irisdaly Estevez, Zachary Marcum, Emma Viscidi

**Affiliations:** Aetion, Inc., New York City, New York; Moderna Therapeutics, Cambridge, Massachusetts; Aetion, Inc., New York City, New York; Aetion, Inc., New York City, New York; Aetion, Inc., New York City, New York; Moderna Therapeutics, Cambridge, Massachusetts

## Abstract

**Background:**

An estimated one in three adults in the United States (US) will develop Herpes zoster (HZ) during their lifetime, with substantially increased risk after age 50. Approximately 15% of individuals with HZ develop postherpetic neuralgia (PHN). HZ is vaccine-preventable, and vaccination is currently recommended for those 50 years of age and older, as well as individuals 19 years of age and older with weakened immune systems. Contemporary data on HZ vaccination, and the burden of HZ and PHN in US adults, is needed.

**Methods:**

This observational cohort study was conducted using administrative medical and pharmacy claims data from HealthVerity and included insured individuals (commercial, Medicare Advantage, or Medicaid plans) across the US. Among patients with continuous enrollment from 01/01/2019 to 05/31/2022, the distribution of patients with 1 and 2 doses of Shingrix (Recombinant zoster vaccine) was estimated. Crude and US age-sex standardized incidence rates of HZ and PHN were calculated from 01/01/2019 to 12/31/2021, by calendar year, in persons >19 years. HZ was defined as >1 ICD-10 diagnosis code for HZ. PHN was defined as >1 ICD-10 diagnosis code for PHN. Analyses were stratified by age, sex, and immunocompromised status.

**Results:**

Among those >50 years (N=25,286,865), 4.3% (N=1,087,066) received 1 dose and 9.0% (N=2,273,827) received 2 doses of Shingrix anytime from 01/01/2019 to 05/31/2022.

The standardized annual incidence rates of HZ from 2019-2021 ranged from 542 to 685 per 100,000 person-years (py) and from 35 to 38 per 100,000 py for PHN. The incidence rates of HZ and PHN were higher among females, older adults, and immunocompromised individuals.

**Conclusion:**

In this analysis in a US claims database, 9% of persons aged 50+ years received 2 doses of the Shingrix vaccine. Herpes zoster and PHN were more frequent among older adults, females, and immunocompromised individuals. This study provides contemporary, representative estimates of the incidence of herpes zoster and PHN, as well as the frequency of herpes zoster vaccination, which is important information on disease burden in the US.

**Disclosures:**

**Alan Embry, PhD**, Moderna Therapeutics: Stocks/Bonds **Brent Arakaki, BS**, Aetion, Inc.: Employee **Emma Viscidi, PhD, MHS**, Moderna: Employee|Moderna: Stocks/Bonds

